# c-MET通路和抑制剂在非小细胞肺癌中的研究进展

**DOI:** 10.3779/j.issn.1009-3419.2017.04.10

**Published:** 2017-04-20

**Authors:** 艳珺 徐

**Affiliations:** 1 310053 杭州，浙江中医药大学 Zhejiang Chinese Medical University, Hangzhou 310053, China; 2 310022 杭州，浙江省肿瘤医院胸部肿瘤内科 Department of Thoracic Oncology, Zhejiang Cancer Hospital, Hangzhou 310022, China

**Keywords:** 肺肿瘤, c-MET, 扩增, 克唑替尼, Lung neoplasms, c-MET, Amplification, Crizotinib

## Abstract

c-MET被认为是继表皮生长因子受体（epidermal growth factor receptor, *EGFR*）基因突变和间变性淋巴瘤激酶（anaplastic lymphoma kinase, *ALK*）基因融合之后，非小细胞肺癌（non-small cell lung cancer, NSCLC）又一个重要的驱动基因。MET的激活包括突变、扩增和蛋白质过表达，是NSCLC潜在的治疗靶点，并提示与预后相关。临床证据表明，MET既可以作为肺癌的原发致癌驱动基因，也是EGFR靶向治疗获得性耐药的原因之一。本文主要对c-MET通路在NSCLC中的活性形式及治疗的研究进展进行综述。

## Abstract (en)

c-MET is considered a promising oncogenic driver in non-small cell lung cancer (NSCLC) after the discovery of epidermal growth factor receptor (EGFR) and anaplastic lymphoma kinase (ALK). MET activation including gene mutation, amplification and protein overexpression, all of these are potential therapeutic targets and are associated with poor prognosis. Clinical evidence suggests a role for MET activation as both a primary oncogenic driver in subsets of lung cancer, and as a secondary driver of acquired resistance to EGFR-tyrosine kinase inhibitor (TKI). This review focuses on the MET activation in NSCLC and the latest trials of its treatment.

自2007年*MET*扩增被发现可能是一代表皮生长因子受体酪氨酸激酶抑制剂（epidermal growth factor receptor-tyrosine kinase inhibitor, EGFR-TKI）的耐药机制之一以来，c-Met通路在非小细胞肺癌（non-small cell lung cancer, NSCLC）中的研究逐渐成为一个热点^[1, 2]^。肝细胞生长因子（hepatocyte growth factor, HGF）/c-Met是一个复杂又独特的信号通路，在正常组织发育和肿瘤发生发展中都起着举足轻重的作用。c-Met参与调控多个生物学功能，包括增殖和侵袭，当失调的c-Met异常激活，可以导致肿瘤的生长和转移。c-Met已逐渐成为新的抗肿瘤治疗靶点。多项临床试验将*MET*抑制剂用于治疗各种实体瘤，特别是在NSCLC中，*MET*抑制剂表现出了一定的疗效。本文将针对c-MET通路在NSCLC中的研究进展及*MET*抑制剂在NSCLC中的临床研究结果作一综述。

## 1 c-MET通路及其异常激活的类型

*MET*基因位于人类7号染色体长臂（7q21-31），长度约125 kb，同时含有21个外显子^[3, 4]^。c-MET是*MET*基因编码产生的具有自主磷酸化活性的跨膜受体，属于酪氨酸激酶受体（receptor tyrosine kinases, RTKs）超家族，由膜外Sema域、PSI域、IPT域和膜内JM域、催化TK域、C末端组成，主要表达于上皮细胞（图 1）。HGF是目前发现的c-MET的唯一配体，属于纤维蛋白溶酶原家族，由N末端、Kringle域、C末端组成，主要表达于间质细胞，亦可表达于肿瘤细胞而通过自分泌机制发挥作用。HGF与c-MET的Sema域结合使c-MET发生二聚、酪氨酸磷酸化，激活众多下游信号通路，如PI3K-Akt、Ras-MAPK、STAT和Wnt/β-catenin等，从而发挥其促细胞增殖、细胞生长、细胞迁移、侵袭血管及血管生成等效应，在组织正常发育和肿瘤进展中发挥关键作用。c-MET通路正常表达时促进组织的分化与修复，当调节异常时则促进肿瘤细胞的增殖与转移。c-MET通路异常激活主要包括*MET* 14外显子跳跃突变、*MET*扩增和*MET*蛋白过表达3种类型。

**1:**
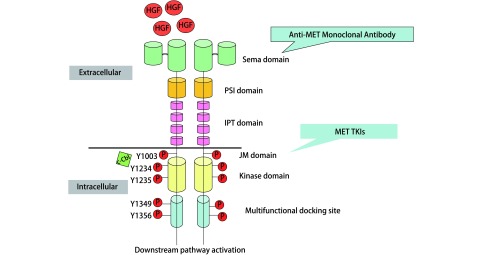
MET通路 MET signialing pathway. Semaphorin (Sema) domain; Plexin-semaphorin-integrin (PSI) domain; Immunoglobulin-plexin-transcription (IPT) domain; Juxtamembrane (JM) domain; TKI: tyrosine kinase inhibitor

### 1.1 *MET* 14外显子跳跃突变

*MET* 14外显子编码部分的JM域，包含Y1003和c-Cbl E3泛素连接酶结合位点。当发生*MET* 14外显子跳跃突变时，Y1003和c-Cbl的结合位点缺失，从而导致受体泛素化降低^[5]^，*MET*蛋白质降解，使*MET*持续激活^[6]^，并作为原发致癌驱动基因。

研究^[7]^报道显示*MET* 14外显子跳跃突变在肺腺癌中发生率约为3%。然而，国内研究^[8]^显示中国肺腺癌中的发生率仅为0.9%，远低于既往研究报道的3%。

*MET* 14外显子跳跃突变形式多样，目前，以DNA为基础的NGS是最常用检测技术^[9, 10]^。另外，*MET* 14外显子跳跃突变常伴有免疫组化（immunohistochemistry, IHC）下的*MET*过表达。因此，可以先用IHC对患者进行筛选，缩小目标人群^[6]^。

*MET* 14外显子跳跃突变不与*EGFR*、*KRAS*、*ALK*等肺癌其他突变共存，提示*MET* 14外显子跳跃突变是原发致癌驱动基因^[11]^。但*MET* 14外显子跳跃突变可与*MET*扩增和MDM2扩增重叠，在NSCLC中与*MET*高扩增并存机率约为3.3%，同时与*MET*蛋白高表达相关^[12]^。当下针对*MET* 14外显子跳跃突变的治疗药物主要是克唑替尼和卡博替尼。

### 1.2 *MET*扩增

*MET*扩增即*MET*拷贝数扩增，包括整体染色体重复和局部区域基因的重复^[13]^。整体染色体重复即多倍体，肿瘤细胞中出现多条7号染色体。有研究^[14]^表明，多倍体扩增通常伴有其他基因突变，如*EGFR*、*KRAS*，这提示多倍体扩增不是驱动基因。*MET*扩增与*EGFR*、*KRAS*或其他驱动基因的激活有明确的联系，是获得性耐药的机制之一^[15]^。

通常使用荧光原位杂交技术（fluorescence *in situ* hybridization, FISH）检测*MET*拷贝数扩增。对于FISH诊断*MET*扩增还没有统一的标准，常用的有Cappuzzo评价系统和PathVysion两种方法^[6]^（表 1）。*MET*高扩增在肺腺癌中发生率为1.0%，在高加索人群和亚裔人群中没有明显差异，提示发生率与人种无关^[12]^。尽管*MET*扩增的发生率不高，但常伴有较强的*MET*蛋白表达，同时也是预后不良的因素之一。*MET*抑制剂对于*MET*高扩增的患者有明显获益^[16]^。约15%-20%的*EGFR*获得性耐药患者可检测到*MET*扩增，*MET*扩增可同时伴有T790M突变或小细胞肺癌（small cell lung cancer, SCLC）转化。*MET*扩增也是三代*EGFR*-TKIs的重要耐药机制之一^[6]^。

**1:** *MET*扩增的评价标准 Quantification criteria for *MET* amplification

	Low-level amplification	Intermediate-level amplification	High-level amplification
Cappuzzo scoring system	MET copy number≥5, < 6	MET copy number≥6, < 7	MET copy number≥7
PathVysion	1.8≤MET/CEP7≤2.2	2.2 < MET/CEP7 < 5	MET/CEP7≥5

### 1.3 *MET*蛋白过表达

许多因素都会引起*MET*激活，如其他致癌驱动基因，缺氧的环境，炎症因子，促血管生成因子和HGF^[4]^。*MET*激活状态中最常见的表现就是转录上调引起的蛋白过表达。但将*MET*蛋白过表达作为激活形式之一目前尚有争议。尽管*MET*蛋白过表达在肺腺癌中的发生率可高达65%^[17]^，但并非作为原发致癌驱动因素，更多的时候是作为其他驱动基因激活后产生的二次事件，从而促进肿瘤的生长^[6]^。

## 2 针对c-MET通路异常激活的治疗策略

针对*MET*或其配体HGF的靶向药物很多，主要分为两大类：单克隆抗体和靶向*MET*基因的小分子TKIs。MET抑制剂又分为多激酶或选择性的*MET*抑制剂。克唑替尼、卡博替尼属于多激酶*MET*抑制剂。选择性*MET*抑制剂包括竞争性ATP抑制剂Capmatinib（ICN280）、tepotinib和非竞争ATP抑制剂tivantinib。单克隆抗体分为*MET*抗体（onartuzumab, emibetuzumab）和抗GF抗体（ficlatuzumab, rilotumumab）^[6]^（表 2）。

**2:** 目前治疗c-MET通路异常药物的临床试验 Latest clinical trials with drugs treating c-MET activation

Agents	Clinical trial	Patients	Methods	Primary endpoint
Crizotinib	PROFILE 1001 Ⅰ/Ⅱ	Advanced NSCLC patients with MET ex14 alterations	Crizotinib 250 mg *bid*	ORR
	(NCT00585195)	Advanced NSCLC patients with MET amplification	Crizotinib 250 mg *bid*	ORR
Cabozantinib	Ⅱ NCT01708954	Advanced EGFR wild-type NSCLC patients with MET IHC-positive	Cabozantinib 40 mg *qd* Cabozantinib 40 mg qd+Erlotinib 150 mg *qd*	PFS PFS
Tivantinib	MARQUEE Ⅲ	Advanced EGFR wild-type non-squamous NSCLC patients	Tivantinib 360 mg *bid*+Erlotinib 150 mg *qd*	OS
	(NCT01244191) ATTENTION Ⅲ (NCT01377376)	Advanced *EGFR* wild-type non-squamous NSCLC patients	Tivantinib 360 mg *bid*+Erlotinib 150 mg *qd*	OS
Capmatinib (ICN280)	Ⅰb/Ⅱ NCT01610336	c-MET+ advanced NSCLC patients with acquired resistance to EGFR-TKI	ICN280 400 mg *bid*+Gefitinib	ORR
Emibetuzumab (LY2875358)	Ⅱ NCT01900652	c-MET+ advanced NSCLC patients with acquired resistance to erlotinib	Emibetuzumab monotherapy Emibetuzumab+Erlotinib 150 mg *qd*	ORR ORR
NSCLC: non-small cell lung cancer; EGFR-TKI: epidermal growth factor receptor-tyrosine kinase inhibitor; IHC: immunohistochemistry; ORR: objective response rate; PFS: progression-free survival; OS: overall survival.				

### 2.1 *MET* 14外显子跳跃突变治疗

在2016年美国临床肿瘤学会（American Society of Clinical Oncology, ASCO）年会上报告了一项关于克唑替尼治疗*MET* 14外显子跳跃突变晚期NSCLC患者的Ⅰ期/ Ⅱ期临床研究^[18]^。入组18例*MET* 14外显子跳跃突变患者，71%为腺癌，18%为肉瘤样癌，6%为腺鳞癌，6%为鳞癌。在15例可评估疗效的患者中，10例患者疗效评价部分缓解（partial response, PR）（67%），其中5例确定PR，另5例未确定PR。中位PFS未达到。不良反应主要有腹泻、恶心、呕吐、周围性水肿和视觉障碍。大部分不良反应为1级-2级，有1例患者因3级水肿停止治疗。该研究显示克唑替尼对于*MET* 14外显子跳跃突变的患者治疗有效，不良反应能耐受。综上，克唑替尼治疗*MET* 14外显子跳跃突变的前景可观。

### 2.2 原发*MET*扩增治疗

#### 2.2.1 克唑替尼

2014年ASCO年会上报告了一项研究克唑替尼治疗*MET*基因扩增的晚期NSCLC的Ⅰ期临床试验^[19]^。该研究共入组13例原发*MET*扩增患者，其中低扩增1例，中扩增6例，高扩增6例，接受克唑替尼250 mg *bid*治疗。结果显示，在可评估的12例患者中，4例患者疗效评价为PR（33%），其中扩增1例（20%），高扩增3例（50%）。中位缓解持续时间为35周，中位治疗持续时间为15.7周。常见不良反应基本与上述一致。综上，克唑替尼用于*MET*扩增前景尚可，并且发现扩增程度与疗效成正相关。

#### 2.2.2 卡博替尼联合厄洛替尼

一项比较卡博替尼，厄洛替尼或双药联合作为二线/三线治疗EGFR野生型晚期NSCLC患者疗效的Ⅱ期临床试验^[20]^入组125例患者，随机分组，最后可评估的为111例。其中使用厄洛替尼单药治疗38例（34%），使用卡博替尼单药治疗38例（34%），卡博替尼联合厄洛替尼治疗35例（32%）。结果显示，与厄洛替尼单药组（PFS 1.8个月）相比，卡博替尼单药（4.3个月）和卡博替尼联合厄洛替尼（4.7个月）均有显著的PFS获益。同时，卡博替尼单药（HR=0.39, *P*=0.000, 3）或双药联合（HR=0.37, *P*=0.000, 3）对总生存期（overall survival, OS）也均有获益。常见3级、4级不良反应主要是腹泻、高血压、乏力、口气黏膜炎、血栓。卡博替尼组1例患者因呼吸衰竭死亡，考虑与药物治疗相关。双药联合组1例患者因肺部感染死亡，考虑与药物治疗相关。综上研究结果，卡博替尼治疗*MET*扩增前景可观。但双药联合毒副作用较重，且PFS与卡博替尼单药相差不大。资料中推荐卡博替尼单药治疗NSCLC中的*MET*扩增。

#### 2.2.3 Tivantinib联合厄洛替尼

MARQUEE^[21]^和ATTENTION^[22]^两项Ⅲ期研究针对晚期EGFR野生型非鳞NSCLC患者，结果都没有达到预期终点，OS均没有获益，提示tivantinib联合厄洛替尼对于*MET*原发扩增无效。尽管两组试验中tivantinib联合厄洛替尼组较厄洛替尼单药组均提示有PFS获益，但没有统计学差异。

#### 2.2.4 MEK抑制剂

日本的一项基础研究提示MEK抑制剂（曲美替尼和PD0325901）对于*MET*扩增的NSCLC细胞系治疗有效。并且当联合*MET*抑制剂（克唑替尼）时，治疗*MET*扩增的NSCLC细胞系疗效特别显著。这项实验结果鼓励我们进一步探讨*MET*抑制剂联合MEK抑制剂治疗NSCLC中的*MET*扩增的可行性研究^[23]^。

### 2.3 EGFR-TKI耐药后*MET*扩增的治疗

由于*MET*扩增是EGFR-TKI治疗出现获得性耐药的原因之一，而且*MET*活化导致的EGFR-TKI耐药，患者的EGFR通路仍然活跃。对于这类患者，若要克服耐药，需要采用EGFR-TKI联合*MET* TKI的治疗策略，相关临床试验应运而生[如Capmatinib（ICN280）联合吉非替尼（NCT01610336）^[24]^、Emibetuzumab（LY2875358）联合厄洛替尼（NCT01900652）^[25]^]。

#### 2.3.1 Capmatinib（ICN280）联合吉非替尼

吴一龙教授牵头的一项Ⅰb期/Ⅱ期临床研究，目前报道了对于EGFR-TKI耐药的cMET+ NSCLC患者，ICN280 400 mg（*bid*）联合吉非替尼治疗具有较好的耐受性，并表现出一定的临床疗效，*cMET*高扩增的患者可能临床获益更大。在2016年ASCO年会上报道了其在*MET*基因异常NSCLC中的疗效^[24]^。该研究Ⅱ期结果显示在83例入组肺癌患者中，66例（80%）曾接受EGFR-TKI单药或联合治疗，42例（51%）中断了治疗，多数（34%）是因为疾病进展。在65例可评价疗效的患者中，12例PR[客观缓解率（objective response rate, ORR）=18%]，40例稳定（SD=62%），疾病总体控制率为80%。在53例IHC 3+或IHC 2+且GCN≥5的肺癌患者中，10例出现部分缓解（ORR=19%）；在23例GCN≥6的肺癌患者中，7例出现部分缓解（ORR=30%）。最常见的不良反应依次为低蛋白血症（29%）、周围性水肿（27%）、食欲减退（23%）。最常见的3级-4级不良反应为淀粉酶升高（7%）。该研究结果提示INC280治疗EGFR TKI耐药后cMET+ NSCLC患者安全有效，值得进一步探索。

#### 2.3.2 Emibetuzumab（LY2875358）联合厄洛替尼

2016年ASCO年会上报道了一项比较emibetuzumab单药或联合厄洛替尼治疗厄洛替尼耐药伴*MET*表达阳性的晚期NSCLC的Ⅱ期临床试验^[25]^中，在IHC测定*MET*表达≥60%的患者中，联合用药组的ORR为3.8%，emibetuzumab单药组的ORR为4.8%。在*MET*表达≥10%的患者中，联合用药组的ORR为3.0%，单药组ORR为4.3%。DCR和PFS都是联合用药组（50%/3.3个月）比emibetuzumab单药组（26%/1.6个月）要好。综上，emibetuzumab治疗厄洛替尼耐药的*MET*+ NSCLC有临床获益，且联合用药的疗效要好于单药，毒副作用可控。

## 3 未来的发展方向

### 3.1 克唑替尼治疗*MET* 14外显子跳跃突变的耐药机制及其治疗策略

#### 3.1.1 耐药机制

有3个病例报道研究克唑替尼治疗*MET* 14外显子跳跃突变的耐药机制^[26-28]^。3例患者均为*MET* 14外显子跳跃突变，一线或二线接受化疗和局部放疗，疾病进展后接受克唑替尼治疗直到疾病再次进展，疗效评价PR。重新进行基因检测，1例患者提示在原有*MET* 14外显子跳跃突变D1010H的基础上，新出现*MET* 19外显子D1228N突变，还有1例患者发现原有的*MET* 14外显子跳跃突变（D1010H）水平降低至10.9%，而*MET* Y1230C突变升至3.5%。另1例患者发现在原有*MET* 14外显子跳跃突变基础上，新发*MET* 19外显子D1228N/H和Y1230H，同时出现3个突变。上述病例提示D1228和Y1230突变可能是*MET* 14外显子跳跃突变患者对克唑替尼产生获得性耐药的主要原因。

#### 3.1.2 耐药后的治疗策略

Klempner等^[29]^报道了1例65岁高加索男性NSCLC患者，Ⅰa期手术，17个月后复发，多发肝转移和2个脑转移病灶。对手术病理组织进行基因检测，发现*MET* 14外显子跳跃突变，*MET*扩增阴性，没有其它驱动基因突变。对脑转移病灶进行SRS放疗后，开始服用克唑替尼，4周后发生肝转氨酶升高至4级，影像检查发现肺部肿瘤缓解，但多发脑转移。肝转氨酶降至1级后，开始服用卡博替尼60 mg每天，4周后复查发现脑转移病灶完全消失，肺部肿瘤持续缩小，肝转氨酶保持正常值。该病例提示，在*MET*原发突变克唑替尼耐药之后，卡博替尼是进一步治疗的选择之一。

### 3.2 *MET*抑制剂联合免疫治疗

免疫治疗已经成为继化疗、放疗、手术、靶向治疗之后的又一种肺癌标准治疗方式。肿瘤突变负荷与pembrolizumab疗效密切相关。有报道显示，可以将肿瘤突变负荷作为抗原的替代标志，当抗原高表达时提示肿瘤负荷较高。在*MET* 14外显子跳跃突变患者中，肿瘤突变负荷平均为6.9个突变/Mb，低于肺癌平均突变负荷10.7个突变/Mb，但高于*EGFR*-突变（平均4.5）和ALK+ NSCLC（平均2.8）。在*EGFR*突变和ALK+ NSCLC患者中，很少会同时伴有PD-L1表达。尽管在*MET* 14外显子跳跃突变的患者中PD-L1表达水平目前还未知，但这些患者的平均肿瘤突变负荷高于EGFR+或ALK+ NSCLC患者，特别是在*MET*扩增亚组中。这提示*MET*抑制剂联合免疫治疗治疗可能会有一定的疗效^[30]^。

## 4 小结

*MET*不仅是EGFR-TKI耐药后的新靶点，亦是原发致癌驱动基因。针对*MET*通路异常激活的几种形式，如何筛选出可能从*MET*抑制剂中获益的患者群体，是目前面临的主要挑战。*MET*抑制剂在*MET*通路异常激活的患者中初显成效，现在最被认可的药物是克唑替尼。但在克唑替尼耐药之后，我们的治疗手段就极其有限。如何应对不可避免要产生的耐药现象，需要更多的药物临床试验和转化研究给我们提供启发。耐药之后，需要再次行基因检测，因此我们可以看到，在实现精准医学的过程中，分子检测非常重要，贯穿了整个疾病的诊治过程。未来，*MET*通路异常激活的NSCLC患者必将会获得更好的疗效和更长的生存期。
